# Intra-day variation in daily outdoor walking speed among community-dwelling older adults

**DOI:** 10.1186/s12877-021-02349-w

**Published:** 2021-07-08

**Authors:** Hisashi Kawai, Shuichi Obuchi, Ryo Hirayama, Yutaka Watanabe, Hirohiko Hirano, Yoshinori Fujiwara, Kazushige Ihara, Hunkyung Kim, Yoshiyuki Kobayashi, Masaaki Mochimaru, Eiki Tsushima, Kozo Nakamura

**Affiliations:** 1 Tokyo Metropolitan Institute of Gerontology, 35-2 Sakae-cho, Itabashi-ku, Tokyo, 173-0015 Japan; 2 Osaka City University, Osaka, Japan; 3 Gerodontology, Department of Oral Health Science, Faculty of Dental Medicine, Hokkaido University, Sapporo, Japan; 4 Faculty of Medicine, Hirosaki University, Aomori, Japan; 5 Human Augmentation Research Center, National Institute of Advanced Industrial Science and Technology, Tokyo, Japan; 6 Towa Hospital, Tokyo, Japan

**Keywords:** Frailty, Global positioning system, Intra-day variation, Smartphone, Walking speed

## Abstract

**Background:**

Walking speed is an important measure associated with health outcomes in older individuals, such as dependency and death. This study aimed to examine whether the walking speed of community-dwelling older adults varies between time periods within a day, as measured outdoors in daily life. We aimed to determine the types of walking speed variations and examine the factors associated with them.

**Methods:**

Daily life outdoor walking speed was measured in 92 participants (average age 71.9 years±5.64) using a GPS smartphone app for 1 month. Average walking speeds for five time periods were analyzed with a linear mixed model. Intra-day walking speed variation patterns were classified by latent class analysis. Factors associated with the class were identified by logistic regression analysis.

**Results:**

A statistically significant difference in average walking speed was found between early morning (1.33 m/s), and afternoon (1.27 m/s) and evening (1.26 m/s) (*p* < 0.01). The intra-day variation in walking speed was attributed to variation in cadence. Two classes were identified: (1) fast walking speed with large variation and (2) slow walking speed with little variation; hypertension and frailty level were associated with the class.

**Conclusion:**

The results suggest that there is intra-day variation in walking speed in daily life, wherein the speed is the fastest early in the morning and slower in the afternoon and evening. A larger variation in the walking speed was related to the health status without hypertension or frailty. These results suggest that if a person shows less intra-day variation in walking speed, this could be a sign that they are susceptible to hypertension and an increased frailty level.

**Supplementary Information:**

The online version contains supplementary material available at 10.1186/s12877-021-02349-w.

## Background

Walking speed is associated with older individuals’ health outcomes. Epidemiological studies have reported a relationship between decreased walking speed in older adults and the impairment of instrumental activities of daily living [1, 2]. Recently, frailty [3] and sarcopenia [4] have been proposed as adverse health outcomes that can affect prognoses for older adults, and walking speed is a diagnostic measure for both concepts. A pooled analysis of large-scale cohort studies has shown that a faster walking speed was related to the survival rate [5]. Walking speed is a basic movement within daily human activities; therefore, a faster walking speed has been recognized as a “vital sign” of good physical health in evaluating older populations’ general health, morbidity, and mortality [6].

In previous epidemiological research and medical checks, walking speed is commonly measured using a stopwatch to determine the time taken to walk on a walkway for a certain distance (e.g., 5 or 10 m) [7]. This method is simple and easy and has been shown to be sufficiently accurate for measuring walking performance [8], and it has been used in numerous studies [7, 9]. However, participants can intentionally change their walking speed when this method is used. Participants can also unintentionally change their walking speeds. For example, the discretization of laboratory-type measures can cause artificial increases in walking speeds [10]; in addition, the presence of the experimenters during data collection also increases the walking speed [11]. While this may have a small effect on maximum performance (i.e., walking a relatively short distance as fast as possible), normal walking speed (i.e., walking at a comfortable pace) measured in this way may somewhat differ from daily life walking speed, and several studies have indicated such differences [12–17]. Measuring the walking speed remotely (without an examiner) in daily life could be a better tool for the long-term assessment of older individuals’ health conditions, because it can evaluate the natural walking speed in daily life and can be measured continuously.

Studies that have measured daily life gait have employed technologies including wearable accelerometer sensors and smartphone apps [12–21]. Recent developments in sensor technology enable the measurement of daily life walking speed. In our previous study [19], we defined daily living walking speed (DWS) as the average of multiple walking speed measurements taken during daily life using a smartphone with a built-in global positioning system (GPS) and showed its re-test reliability. Moreover, we presented age-related changes in DWS [20] and the association between DWS and physical function and pre-frailty [15]. Those studies indicated that DWS declines with age, like walking speed measured in the laboratory, and that discriminability of pre-frailty is comparable to laboratory walking speed, suggesting that DWS can be utilized for physical health assessment. However, there is no standard consensus on the definition of DWS, representative values, or measurement methods. Although studies related to DWS have increased in recent years [13, 14, 18, 21], its definition and measurement methods are varied, and the definition of measurement of DWS has not been established. Therefore, research related to the definition of DWS is needed.

Walking speed is influenced by place (e.g., shopping centers or residential areas) [22], noise [23], temperature [24], and season [25]. A previous study reported that there were no within-day variations for walking speeds measured in a laboratory setting [26]. Although one study on outdoor walking (without an examiner) did not analyze the variations in individual participants’ walking speed, it suggested differences in walking speed in accordance with time periods within a day [27]. Changes in daily walking speed according to time of day may be as important as average walking speed values measured in daily life similar to the heart rate variability [28]. However, no studies have investigated intra-day walking speed variation based on measured values.

Thus, this study aimed to examine whether the walking speed of community-dwelling older adults varies between time periods within a day, measured outdoors in daily life using smartphones with built-in GPS. Furthermore, if speeds vary, we aimed to determine the types of variations and examine the factors associated with them.

## Methods

### Participants

**Fig. 1:**
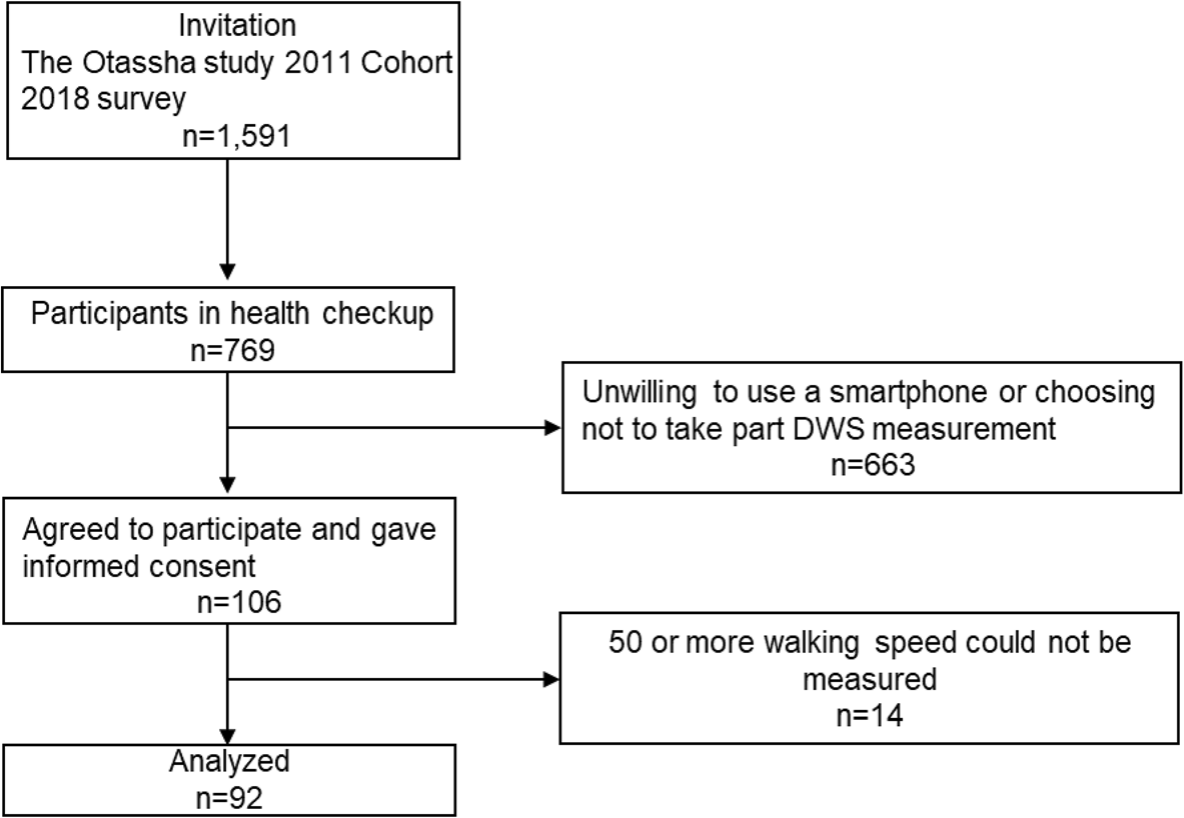
Flow of participants through study

This study’s participants were recruited in 2018 from the comprehensive health survey, “The Otassha Study 2011 Cohort.” This cohort targeted community-dwelling older adults, and a notification letter was mailed in 2011 to all 6699 residents aged 65–84 years living in the nine areas of Itabashi Ward, Tokyo [29]. This baseline survey was administered at the survey location and included 913 individuals. Past participants and new 65-year-old ones have been invited to follow-up surveys every year. The 2018 survey was conducted between 1 and 8 October with 769 participants (Fig. 1). At the survey location, participants were asked whether they would participate in DWS measurement using a smartphone app for 1 month; 106 participants expressed interest [15]. Reasons for non-participation included being unwilling to use a smartphone or choosing not to take part.

Participants were requested to install the walking speed measurement app (Chami, InfoDeliver Co. Ltd., Tokyo, Japan) on their smartphones, carry their smartphones when they left their homes as much as possible, and conduct their daily lives as normal for 1 month starting from the day after the survey was administered. Smartphones pre-installed with the app were lent to participants who did not own smartphones.

This study was conducted in accordance with the 1964 Declaration of Helsinki and its later amendments. The study was approved by the ethics committee of the Tokyo Metropolitan Geriatric Hospital and Institute of Gerontology (no. K120; 2018). Informed consent was obtained from all participants.

### Walking speed measurements

The smartphone app measuring walking speed employed a step counter and GPS. The authors’ previous research showed this technique to have DWS re-test reliability and the validity of stopwatch measurement, which is the gold standard [19]. The app used GPS to identify where the participant’ walked and, when a stable walking trajectory was detected, it measured walking speed until the participant stopped. We defined stable walking as a subject’s walking trajectory of 20 m or more detected using the linear least squares method from position information acquired by GPS. The smartphone-equipped step counter function was used to detect when the participant was walking. The walking speed was calculated by dividing the traveled distance over the stable walking trajectory by the elapsed time. Measurements were performed automatically, and no additional operations were required by the participant, making it possible to measure walking speed without participants’ awareness. The data for each continuous walk were sent to the data server. The walking speed data, including the time of measurement, the number of steps, and the distance of the walk, were stored on a dedicated server.

The use of GPS implies that measurements were limited to outdoor walking. While there were differences due to participants’ lifestyles, approximately 50 to 1000 walking speed measurements were recorded per participant during the one-month period. Walking speeds measured this way may include various types of walking, including strolling, walking exercise, hurried walking, and walking with a companion, or the type of terrain, which could affect the walking speed. In our previous studies [19, 20], we confirmed that 50 or more walking speeds measured in daily life have a unimodal normal distribution and that the average value is representative of typical daily walking speeds. We believed to be able to calculate the representative walking speed for each time period by measuring a certain number of continuous walks, including multiple steps over 1 month.

### Physical frailty assessment

To investigate intra-day walking speed variation from the perspective of physical frailty, this study employed the diagnostic criteria in the Japanese version of the Cardiovascular Health Study [30] (weight loss, weakness, slowness, exhaustion, and low activity), which were measured at the survey location to evaluate participants’ physical frailty. These criteria are modified from Fried and colleagues [31] to include a threshold for Japanese older adults. The prevalence of frailty has been reported to be similar to conventional criteria in a previous study [30]. Well-trained examiners measured the following items.

“Weight loss” corresponded to the answer “Yes” to question #11 of the Kihon Checklist: [32] “Have you lost more than 2–3 kg in the last six months?” “Weakness” meant the participant’s grip strength was under 26 kg for men or under 18 kg for women. Grip strength was measured once using a Smedley-type dynamometer (AS ONE Corporation, Osaka, Japan) with the participant’s dominant hand. “Slowness” corresponded to a normal walking speed under 1.0 m/s. “Normal walking speed” was measured using a stopwatch to measure the time taken to walk the five-meter walkway with three-meter acceleration and deceleration areas before and after the walkway. Walking speed was measured once. “Exhaustion” corresponded to the answer “Yes” to question #25 of the Kihon Checklist: “(In the last two weeks) I have felt tired for no particular reason.” “Low activity” referred to participants who did not regularly engage in light exercise or sports activity.

Participants with one or two of the above items applied were categorized as “Pre-frail,” participants with three or more as “Frail,” and participants with none of the items as “Robust.”

### Data analyses

Of the collected data, 92 participants with 50 or more walking speed measurements were selected. The characteristics (sex, age, body height, body weight, chronic diseases, grip strength, and normal walking speed) of the participants were compared among frailty categories using t-tests or chi-square tests. The time from 4 am to 11 pm was divided into early morning (4–7 am), morning (8–11 am), afternoon (12–3 pm), evening (4–7 pm), and night (8–11 pm). The number of measurements, average walking speed, step length, and cadence in each period were examined using a linear mixed model and Bonferroni post-hoc test.

A latent class analysis was performed with walking speed for each period as the observed variable to classify variation patterns in walking speed by period. In the latent class analysis, missing data for walking speed in each period were complemented, and the optimal class model was determined based on multiple fit indices (i.e., Akaike information criterion, sample size adjusted Bayesian information criterion, Lo–Mendell–Rubin’s adjusted likelihood ratio test) [33]. Differences in walking speed between the time periods among the classes was derived from the latent class analysis using a linear mixed model and Bonferroni post-hoc test. This study needed a sample size of *n* = 21 for repeated analysis with five measurements and a statistical power of 0.8.

To identify factors associated with the types of variations, logistic regression analysis with the class as a dependent variable, and sex, age, chronic diseases, and frailty level as independent variable, were performed. The latent class analysis was performed with Mplus version 7.4 (Muthén & Muthén, Los Angeles, CA, USA); all other statistical analyses were performed with SPSS version 26.0 (IBM Japan, Ltd., Tokyo, Japan).

## Results

Of the 92 participants, 57 were female (62.0%), and the average age was 71.9 (SD = 5.64). Further, 30 participants were categorized as “pre-frail,” 62 as “robust,” and none as “frail” (Table 1). There were no significant differences for age, sex, body characteristics, and prevalence of chronic diseases between robust and pre-frail participants, but grip strength and normal walking speed were significantly higher in the robust than in the pre-frail participants (Table 1).

**Table 1:** Characteristics of the participants

	Robust (*n* = 62)		Pre-frail (*n* = 30)		*p*†
	Mean	SD	Mean	SD	
Age (years)	72.1	5.43	71.3	6.10	0.520
Height (cm)	158.8	9.76	156.8	8.20	0.330
Weight (kg)	57.5	10.87	57.5	13.13	0.999
Grip strength (kg)	29.7	8.19	24.1	6.78	**0.002**
Normal walking speed (m/s)	1.46	0.217	1.35	0.200	**0.029**
Female (n, %)	36	58.1	21	70.0	0.269
Hypertension (n, %)	25	40.3	13	43.3	0.783
Stroke (n, %)	3	4.8	2	6.7	0.717
Heart disease (n, %)	8	12.9	7	23.3	0.204
Diabetes (n, %)	4	6.5	3	10.0	0.547

†t-test or chi-square test. Bold: *p* < 0.05

The number of measurements during early morning and night were significantly lower than those of the morning, afternoon, and evening (*p* < 0.01) (Table 2). Walking speed in the early morning was significantly faster than in the afternoon (*p* < 0.01) and evening (*p* < 0.01). There were no significant differences for step length between all time periods. Cadence in the early morning was significantly higher than in the afternoon (*p* < 0.01) and evening (*p* < 0.05).

**Table 2:** Walking parameters measured in daily life over four time periods

	**EM**			**MO**			**AF**				
	**n**	**Mean**	**SD**	**n**	**Mean**	**SD**	**n**	**Mean**	**SD**		
Number of measurements	64	59	84	90	100	86	92	128	144		
Walking speed (m/s)	64	1.33	0.16	90	1.29	0.13	92	1.27	0.11		
Step length (m)	64	0.68	0.06	90	0.67	0.06	92	0.67	0.05		
Cadence (step/min)	64	116.87	8.99	90	116.55	7.33	92	113.75	5.57		
	**EV**			**NI**			**Significant differences†**				
	**n**	**Mean**	**SD**	**n**	**Mean**	**SD**					
Number of measurements	92	88	74	75	30	69	EM < MO,AF; MO > NI, AF > EV,NI; EV > NI				
Walking speed (m/s)	92	1.26	0.12	75	1.28	0.16	EM > AF,EV				
Step length (m)	92	0.67	0.05	75	0.67	0.07					
Cadence (step/min)	92	114.16	6.66	75	115.81	8.37	EM > AF,EV				

†*p* < 0.05: Linear mixed model and Bonferroni post-hoc test

*EM* Early morning, *MO* Morning, *AF* Afternoon, *EV* Evening, *NI* Night

**Fig. 2:**
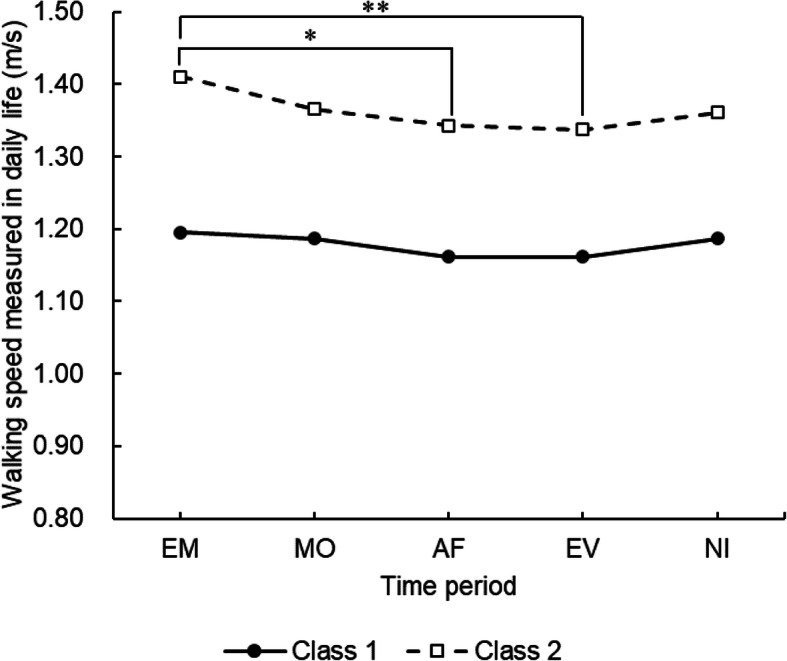
The two classes of intra-day variation in terms of walking speed that were measured in daily life. EM: early morning, MO: morning, AF: afternoon, EV: evening, NI: night; *p < 0.05, **p < 0.01 (linear mixed model and Bonferroni post-hoc test)

The latent class analysis revealed two classes of intra-day variation in walking speed (Fig. 2). There were no significant differences for walking speed between all time periods in Class 1. Class 2 early morning walking speed (1.41 m/s) was statistically significantly faster than afternoon (1.34 m/s, *p* < 0.01) and evening (1.34 m/s, *p* < 0.05) walking speeds. The average walking speed among all time periods in Class 1 was 1.18 m/s, whereas in Class 2 was 1.36 m/s.

As the result of logistic regression analysis to identify the factors associated with the class of variation in the waking speed, sex, age, and hypertension were significantly associated with the class (*p* < 0.01) (Table 3). Frailty level was marginally significantly associated with the class (*p* = 0.059).

**Table 3:** Factors associated with the class of variation of walking speed in daily life

Independent variables	OR	95%CI			*p*†
Sex (Female)	9.289	2.404	–	35.898	**0.001**
Age	1.153	1.038	–	1.280	**0.008**
Hypertension	14.345	3.753	–	54.824	**< 0.001**
Stroke	2.665	0.191	–	37.124	0.466
Heart disease	0.510	0.091	–	2.862	0.445
Diabetes	1.796	0.245	–	13.153	0.564
Frailty (Pre-frail)	3.150	0.959	–	10.343	*0.059*

†Logistic regression analysis; Bold: *p* < 0.05, Italic: *p* < 0.1

Dependent variable: Class of variation of walking speed (reference: class2)

*OR* Odds ratio, *CI* Confidence interval

## Discussion

An intra-day variation of walking speed (fastest walking speed in the early morning, slower walking speed in the afternoon and evening) was suggested. Since an intra-day variation was also observed for cadence, the intra-day variation in walking speed was attributed to an increase and decrease of cadence. The number of measurements was significantly lower in the early morning and night than during the other time periods, indicating that some participants did not walk in the early morning and night. However, the data obtained from all time periods showed an intra-day variation in the walking speed and cadence. Additionally, the latent class analysis revealed two patterns of intra-day variation, with the characteristic of walking speed being fastest in the early morning and slower in the afternoon and evening as particularly remarkable in the class where walking speed was faster. A larger variation of walking speed was related to health status without hypertension or frailty.

Studies related to DWS have increased in recent years. Kiselev and colleagues [21] measured individuals’ daily average walking speeds for up to 10 days using an accelerometer, and Schimpl and colleagues [18] defined it as real-world walking speed. Van Ancum and colleagues [14] measured individuals’ daily walking speeds with an inertial sensor for seven consecutive days, investigating the correlation between each percentile value of daily walking speed and 4-m walking speed measured in the laboratory. Takayanagi and colleagues [13] showed that the daily walking speed measured by an accelerometer for over 7 days was slower than the laboratory walking speed. However, to the best of our knowledge, no other study has investigated intra-day variations in walking speed in daily life within the same participants. A previous study that calculated natural walking speed in daily life from video recorded outdoors investigating the differences between morning, afternoon, and night [25], found that walking speed was fastest in the morning, slower in the afternoon, and even slower at night. However, as that study was conducted prior to the development and widespread dissemination of wearable sensors, it had many limitations, including small sample sizes for older adults and for night, different sample sizes for each period, and only estimated participant ages. The current study is the first to measure natural daily walking speed over several time periods and to show intra-day variations.

There are three possible reasons for fastest walking speed in the early morning that slows in the afternoon and evening. The first is that increased walking speeds in the early morning are due to active older adults engaging in “walking exercise” for health. Research on sports activities in Japan has shown that approximately 70% of older adults engage in such activity [34]. Participants in this study took part in a cohort study with a survey administered at a particular location, and they expressed interest in having their DWS measured with a smartphone. Therefore, this study might have included many relatively active and health-conscious older individuals, who walked fast in the walking exercise and whose walking speed was faster in the early morning.

The second is that participants walked faster in the early morning when they were well rested and less likely to be fatigued than in the afternoon and evening. A previous study reported that fatigue was associated with a slow walking speed [35]. If a participant is walking and exercising all day, they would start to fatigue, and this may influence their walking speeds later in the day.

The third is that cadence is regulated by air temperature. Kimura and colleagues [23] found seasonal variations in walking speed, with winter speeds faster than summer speeds in a lab-based study of older individuals. In their study, changes in the walking speed between summer and winter in the same individuals were examined without adjusting for the room temperature. They suggest that this variation is specific to older adults, with increases in activity speed as a response to lower body temperature in winter. Since our study was conducted in October, the fact that walking speed was fastest in the early morning and slower in the afternoon and evening may be due to air temperature. Moreover, this intra-day variation in walking speed was caused by the change of cadence. Indeed, the average temperature of each period in Tokyo, when the current data were collected in October 2018 [36], was associated with each time period’s average walking speed and cadence (Supplementary Figure 1). Although the reaction of the central walking rhythm control to the temperature may change cadence and cause walking speed to fluctuate, future studies using more detailed data will be necessary to establish an association between temperature and walking speed.

In the latent class analysis of variation patterns, the intra-day variation in Class 2 (faster walking speed) was remarkable. Although a faster walking speed must be an important indicator of good physical health [6], factors associated with the variation class were age, hypertension, and frailty level, suggesting that changes in diurnal variation of walking speed or cadence may also be useful for assessing the risk of disease and frailty. If a person shows less intra-day variation in the walking speed or if they have slower walking speeds, this could be a sign that they have other health-related issues, such as hypertension or frailty. Hypertension in older adults has been reported to be related to adverse cardiovascular outcomes and death [37]. Frailty can be defined as a manifestation of accumulations of physiological dysfunction [38]. Frailty is common in individuals with hypertension [39]. Decreased physical activity and decreased motor function caused by prodromal symptoms of serious diseases and dependency may affect the regulation of the walking speed and cadence in daily life. Walking speed or cadence variation in daily life could be the best predictor of adverse health outcomes, such as disease and frailty.

### Limitations

This study is limited in that it did not include frail participants; walking speeds in daily life of frailer older adults may present variation patterns with specific characteristics. Since this study used smartphone GPS to measure walking speed, the participants were healthy older individuals, who could use a smartphone; they were more active than most older individuals, which may have influenced the increase in the walking speed in the early morning. Additionally, data were only obtained outdoors due to using GPS. If data were collected indoors, variations may be different from those found in the current study. Indoor walking may be more important for severely frail older adults because indoor walking accounts for a large proportion of their daily life. In addition, GPS location estimation errors could be related to the intra-day variation in walking parameters [40]; however, the changes observed in the walking speed were more dependent on changes in cadence and, thus, were not likely affected by GPS errors.

Furthermore, this study did not examine participants’ physical activity, exercise habits, reasons for walking (going out, exercising, shopping, strolling, etc.), the participants’ walking terrain (i.e., slippery terrain, tripping hazards, navigate obstacles, etc.), or body temperatures. Confirmation of these results using such measurements will be necessary.

## Conclusions

This study suggested that there is an intra-day variation in walking speed and cadence in daily life and that a larger variation in the walking speed was related to the health status without hypertension or frailty. These results suggest that if a person shows less intra-day variation in the walking speed, this could be a sign that they are susceptible to hypertension and increased frailty level. Walking speed in daily life can be measured over the long-term and in various situations, and its variation could be used to assess the health status in daily life. Future studies should examine the association of short- and long-term variations in walking parameters of daily life and various health outcomes.

## Supplementary Information

**Additional file 1: Supplementary Figure 1.** Association of average temperature in Tokyo (for October 2018) with the walking speed and cadence in each time period. EM: early morning, MO: morning, AF: afternoon, EV: evening, NI: night; r: Pearson’s correlation coefficient. (LOG 63 bytes).

## Data Availability

The datasets generated during and/or analyzed during the current study are not publicly available because of the inclusion of data from a cohort study in Itabashi Ward containing sensitive participant information and because of ethico-legal restrictions imposed by the Ethics Committee at Tokyo Metropolitan Institute of Gerontology; however, the datasets are available from the corresponding author on reasonable request.

## Abbreviations

DWS: Daily living walking speed
GPS: Global positioning system
