# Supplementary material for: Intra-day variation in daily outdoor walking speed among community-dwelling older adults
Source: BMC Geriatr. 2021 Jul 8;21:417. doi: 10.1186/s12877-021-02349-w (PMC8268528; doi:10.1186/s12877-021-02349-w)
Supplement: Supplementary file 1 — Additional file 1: Supplementary Figure 1. Association of average temperature in Tokyo (for October 2018) with the walking speed and cadence in each time period. EM: early morning, MO: morning, AF: afternoon, EV: evening, NI: night; r: Pearson’s correlation coefficient. (LOG 63 bytes). [file 12877_2021_2349_MOESM1_ESM.docx]

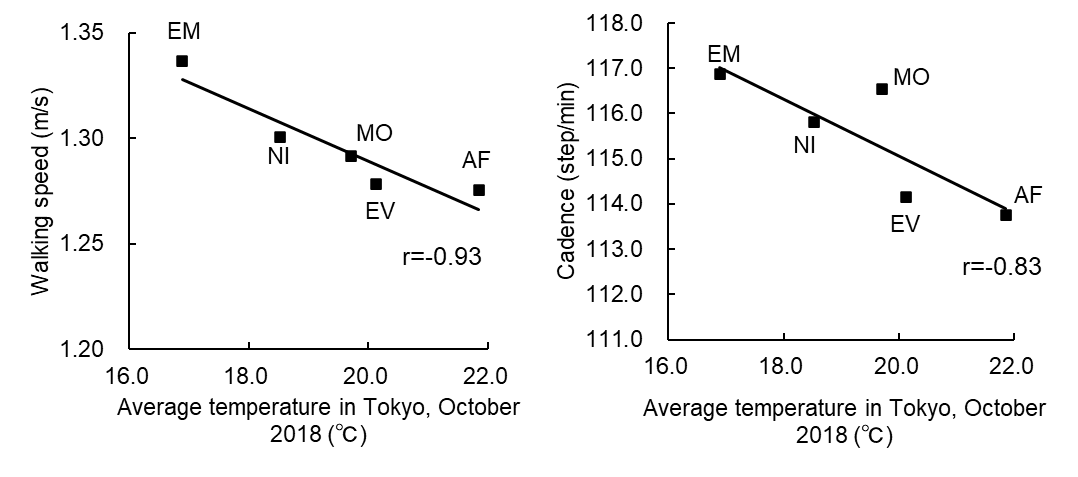


**Supplementary Figure 1.** Association of average temperature in Tokyo (for October 2018) with the walking speed and cadence in each time period.

EM: early morning, MO: morning, AF: afternoon, EV: evening, NI: night; r: Pearson’s correlation coefficient.
